# *Cerophytum lii* Qiu & Ruan, sp. nov. (Coleoptera: Cerophytidae): First Record of the Family in China with Study on Its Thoracic Functional Morphology Through 3D Reconstruction †

**DOI:** 10.3390/insects16090941

**Published:** 2025-09-07

**Authors:** Lu Qiu, Lei Liu, Yongying Ruan, Yang Liu, Liya Ma, Bo Feng

**Affiliations:** 1 School of Life Sciences (School of Ecological Forestry), Mianyang Teachers’ College, Mianxing West Road, Mianyang 621000, China; 123church@163.com; 2 School of Biological and Pharmaceutical Sciences (School of Advanced Agriculture Sciences), Mianyang Teachers’ College, Mianxing West Road, Mianyang 621000, China; 3 Plant Protection Research Center, Shenzhen Polytechnic University, Shenzhen 518055, China; 15222986176ll@gmail.com (L.L.); m15102993050@163.com (L.M.); 4 Key Laboratory of Resource Biology and Biotechnology in Western China (Ministry of Education) and College of Life Science, Northwest University, Xi’an 710069, China; liuyangent@nwu.edu.cn; 5 Department of Entomology, University of Manitoba, Winnipeg, MB R3T 2N2, Canada

**Keywords:** new record, new species, taxonomy, distribution, Elateroidea, rare click beetles

## Abstract

The beetle family Cerophytidae is reported from China for the first time, based on the discovery of *Cerophytum lii* Qiu & Ruan, **sp. nov.** and *Cerophytum* sp., both from Daweishan Mountain in Southeast Yunnan Province. Detailed descriptions and diagnostic illustrations of *Cerophytum lii* **sp. nov.** are provided, along with an identification key to extant *Cerophytum* species worldwide. A representative specimen of the new species (a male paratype) underwent micro-computed tomography scanning, enabling three-dimensional reconstruction and virtual dissection of its thoracic morphology. Internal muscular architecture and exoskeletal structures of its thorax are illustrated and described. Comparative morphological analyses revealed convergent evolution in the clicking structures of *Cerophytum lii* **sp. nov.** and *Campsosternus auratus* (Drury), with the latter exhibiting more specialized structural modifications that enhance its jumping performance to a greater degree. In contrast, *Cerophytum lii* **sp. nov.** exhibits morphological adaptations primarily suited for rapid walking and flight.

## Simple Summary

Cerophytidae, or rare click beetles, is a small beetle family comprising four genera and 23 known modern species. Although relatively common in Europe, Africa, and North America, these beetles are extremely rare in Asia, with only one species previously recorded from Japan and South Korea. Here, we report the first occurrence of Cerophytidae in China with the discovery of a new species, *Cerophytum lii* sp. nov. To better understand how these beetles click and move, we examined the thoracic muscles and exoskeleton of *C. lii* and compared them with *Campsosternus auratus*, a well-known click beetle from the family Elateridae. Our results indicate that *C. lii* is less specialized for jumping but well adapted for rapid walking and flight, whereas *C. auratus* has more specialized structures for jumping. These findings provide new insights into the functional morphology and diversity of rare click beetles.

## 1. Introduction

Cerophytidae (rare click beetles) is an elateroid beetle family comprising 23 extant species classified into four genera: *Cerophytum* Latreille, 1809, *Brachycerophytum* Costa et al., 2003, *Phytocerum* Costa et al., 2003, and *Afrocerophytum* Costa, Vanin and Rosa, 2014 [1,2,3]. Most species of this small family are distributed in the Neotropics, with two species in *Brachycerophytum* and 16 in *Phytocerum* [1,4]. In addition, the monotypic genus *Afrocerophytum* is found in Africa [2,5], while the type genus *Cerophytum*, with four species, has a Holarctic distribution, spanning Europe, Asia, and North America [1]. Members of this family are characterized by their serrate to pectinate antennomere 3–10, elongated metatrochanters, closely spaced antennal sockets, distinctive prosternal process, unique propleurocoxal mechanism, absence of lateral carinae on the prothorax, lack of metacoxal plates, serrate tarsal claws, and a peculiar “dorsal anterior projection of the parameres”, considered to be a “phallobase” in the aedeagus [1,3]. The phylogenetic position of Cerophytidae remains uncertain, although recent studies suggest a close relationship with Throscidae [6,7] or with the recently discovered paedomorphic Jurasaidae from Brazil [8].

The genus *Cerophytum* currently includes four valid species: two from Neotropics (*Cerophytum convexicolle* LeConte, 1866 and *Cerophytum pulsator* (Haldeman, 1845)), one from Europe (*Cerophytum elateroides* (Latreille, 1804)), and one from Japan and South Korea (*Cerophytum japonicum* Sasaji, 1999) [1,9]. In addition, the genus has been recorded in the Russian Far East, although the species identity remains uncertain (*Cerophytum* aff. *japonicum*) [10]. To date, *Cerophytum japonicum* is the only species of Cerophytidae known from Asia, and none has been recorded from China. In this study, we describe a new species of *Cerophytum* from Daweishan, Yunnan, China, establishing the record of the family Cerophytidae in the country. Additionally, a single female *Cerophytum* specimen, representing an undescribed species, is also recorded from Daweishan for future study. The discovery increases the total number of known Coleoptera families in China to 145 [11].

The clicking mechanism and functional morphology of elateroid beetles have been well studied (e.g., [12,13]). The most recent rigorous study is that of Ruan et al. [13], which focuses on the clicking movement and thoracic morphology of the click beetle *Campsosternus auratus* (Drury). Elateridae species are characterized by the prominently enlarged prosternum and mesonotum, along with highly developed M4 muscles, to perform the clicking action [13]. Adult Cerophytidae exhibit clicking behavior analogous to that of Elateridae [14], earning them the designation of “rare click beetles”. However, the functional morphology of the thorax in Cerophytidae remains poorly known. In this study, we investigate the thoracic morphology of *C. lii* sp. nov., through a comparative analysis with *Campsosternus auratus* (Drury).

## 2. Materials and Methods

### 2.1. Specimens

A total of four specimens of the genus *Cerophytum* were collected using light traps in Yunnan Province, China. The studied materials are stored in the Invertebrate Collection of Mianyang Teachers’ College, Mianyang, China (MYTC/MYNU), the Plant Protection Research Center, Shenzhen Polytechnic University, Shenzhen, China (SZPU/SZPT), and the personal collection of Yi-Teng Li (CYTL). The collected data of the studied specimens are presented in English, with the Chinese translations in square brackets.

The body length of specimens was measured from the anterior margin of the head to the apex of the elytra, pronotal length was measured along the mid-line; pronotal width was measured at its widest point; and body width was measured at the widest part of the elytra.

### 2.2. Morphological Terminology

The generic concept of *Cerophytum* follows that of Costa et al. [1]. Morphological terms are based on Ruan et al. [13], and muscles’ names follow those of Larsén [15]. Abbreviations and terms used in the text and Figures 6–8 are listed and explained below.

***3. Pm-ML/3Pm-LP** = median lobe/lateral process of the third phragma; **Abd** = abdomen; **Act** = acetabulum; **AmE** = anteromedium emargination of the mesonotum; **AR** = anterolateral region of the mesonotum, with a highly smooth surface; **PaBr** = prealar bridge of the mesonotum, also known as the prealar arm in Matsuda et al. [16]; **AVA** = anteroventral angle of the mesoventral cavity [17]; **AWP2** = anterior notal wing process of the mesonotum; **AxC** = axillary cord; **BaD** = basalar disc; **Crpl** = cryptopleuron, equivalent to the endopleuron; **Cv1/Cv2** = cervical sclerite 1/2; **Cx1/Cx2/Cx3** = pro-/meso-/metacoxa; **CxP** = metacoxal plate; **Ely** = elytron; **Em2/Em3** = mesepimeron/metepimeron; **Es2/Es3** = mesanepisternum/metanepisternum; **F1** = prothoracic furca; **F2** = mesothoracic furca, equivalent to the mesendosternite; **F3** = metathoracic furca, equivalent to the metendosternite; **FB** = profurcal base or prosternal furcal base, also known as the “bumper” in Evans et al. [12]; **FH** = friction hold in Elateridae, a lowered area on the posterodorsal end of the prosternal process, also known as the “peghold” in Evans et al. [12]; **H** = head; **Hy** = hypomeron; **I** = insertion of muscle; **IAM** = inflected anterior margin of the mesonotum; **LA** = lateral arm of the furca [18]; **LC** = lateral carina [19]; **PRM** = prosternal rest of the mesoventrite, i.e., the anteromedian extension of the meso-ventrite, also known as the “mesosternal lip” and “lip of the mesosternum” in Evans et al. [12]; **M1/M2…M85** = muscles 1–85 [15]; **MAr** = median-arched area of the mesonotum [13]; **MRMs** = median ridge of the metaventrite; **MsC** = mesoventral cavity, also known as the “mesosternal cavity” and the “mesosternal fossa” [17]; **N I/N II/N III** = pro-/meso-/metanotum; **O** = origin of muscle; **PA** = posterior angle of the pronotum; **PdE/PvE** = posterodorsal/posteroventral evagination of the pronotum [13]; **PGr** = posterodorsal groove of the pronotum, situated above the posterodorsal evagination [13]; **PlA** = pleural arm of the meso-/metapleuron; **PlR** = pleural ridge of the meso-/metapleuron; **PmPr** = posteromedial part of the pronotum [13]; **Pn3** = postnotum of the metathorax; **PP** = prosternal process; **Pra** = prealar sclerite of the metathorax, consisting of an externally visible isolated sclerite and the internal mushroom-shaped plate; **Prs3** = metathoracic prescutum; **PsS** = pronotosternal suture; **Sa** = subalar sclerite; **Scl2/Scl3** = meso-/metascutellum; **SclS2** = mesoscutellar shield; **St I** = prosternum; **Stk** = stalk of the metathoracic furca [18]; **Vt II/Vt III** = mesoventrite/metaventrite, known as the mesosternum/metasternum in earlier works; and **YP** = yoke plate [16].*

### 2.3. Dissection Methods and Imaging

The studied specimens were cleaned with warm water, and then the genital segments were dissected after treatment in 10% KOH (70–80 °C for 9 min).

**Figure 1:**
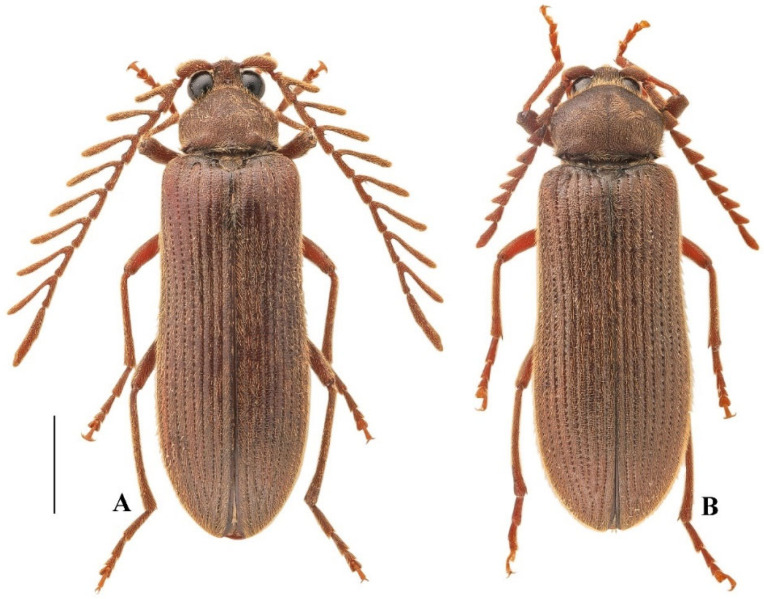
Habitus of *Cerophytum lii* Qiu & Ruan, **sp. nov.**: (**A**) Male holotype, dorsal view; (**B**) Female paratype, dorsal view. Both from Mount Daweishan, China. Scale bar = 2 mm.

**Figure 2:**
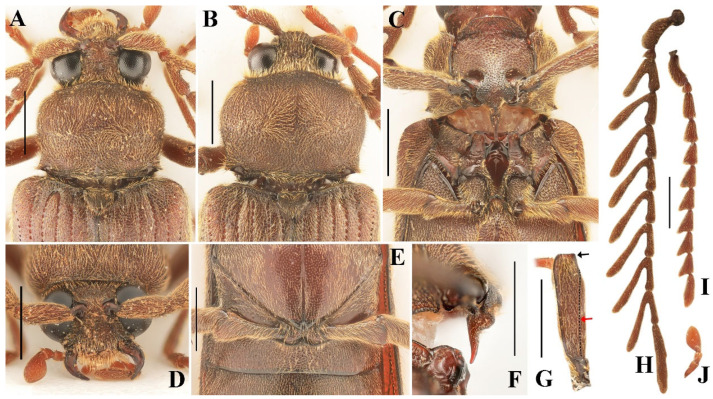
Characteristics of *Cerophytum lii* **sp. nov.**, male holotype (**A**,**C**–**H**,**J**) and female paratype (**B**,**I**), from Mount Daweishan, Yunnan, China: (**A**,**B**) Head, pronotum and scutellar shield, dorsal view; (**C**) Prothorax and mesothorax, ventral view; (**D**) Head, frontal view; (**E**) Metatrochanters, ventral view; (**F**) Prosternal process, lateral view; (**G**) Profemur, posterior view, black arrow indicates the shape of the upper distal angle; red arrow indicates the longitudinal carina; (**H**) Antennae, male; (**I**) Antennae, female; (**J**) Maxillary palpomere I–III. Scale bars = 1 mm.

**Figure 3:**
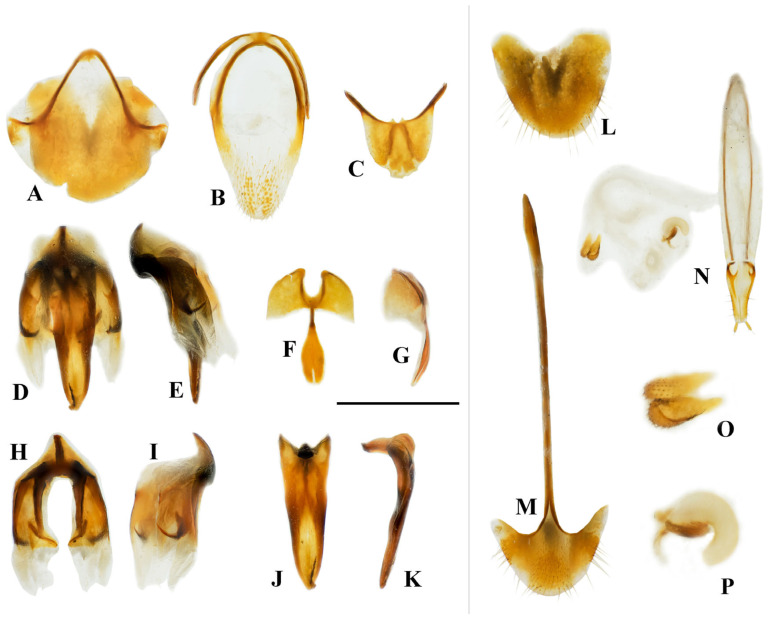
Characteristics of *Cerophytum lii* **sp. nov.**, male holotype (**A**–**K**) and female paratype (**L**–**P**), from Mount Daweishan, Yunnan, China: (**A**) Tergite VIII, ventral view; (**B**) Sternite IX, ventral view; (**C**) Tergite IX + X, dorsal view; (**D**) Aedeagus, ventral view (phallobase removed); (**E**) Aedeagus, lateral view (phallobase removed); (**F**) Phallobase, ventral view; (**G**) Phallobase, lateral view; (**H**) Parameres, dorsal view; (**I**) Parameres, lateral view; (**J**) Median lobe, dorsal view; (**K**) Median lobe, lateral view; (**L**) Tergite VIII, dorsal view; (**M**) Sternite VIII, ventral view; (**N**) Ovipositor and genital tract, ventral view; (**O**) Sclerites of bursa copulatrix; (**P**) Spermatheca. Scale bar = 1 mm for (**A**–**N**); (**O**,**P**) not to scale.

**Figure 4:**
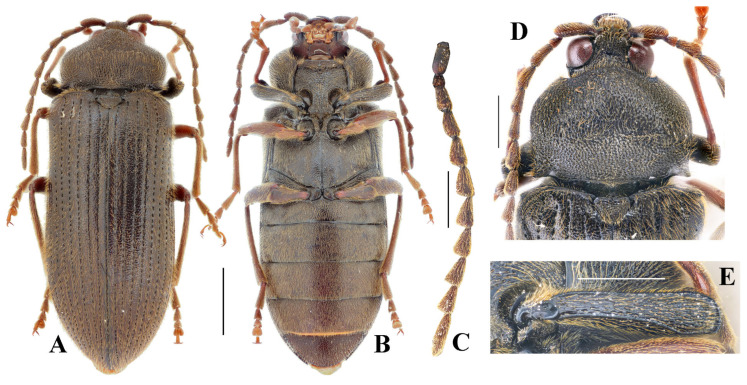
*Cerophytum* sp. from Mount Daweishan, Yunnan, China, female: (**A**) Dorsal view; (**B**) Ventral view; (**C**) Antenna; (**D**) Head, pronotum and scutellar shield, dorsal view; (**E**) Profemur, posterior view. Scale bars: (**A**,**B**) = 2 mm, (**C**–**E**) = 1 mm.

**Figure 5:**
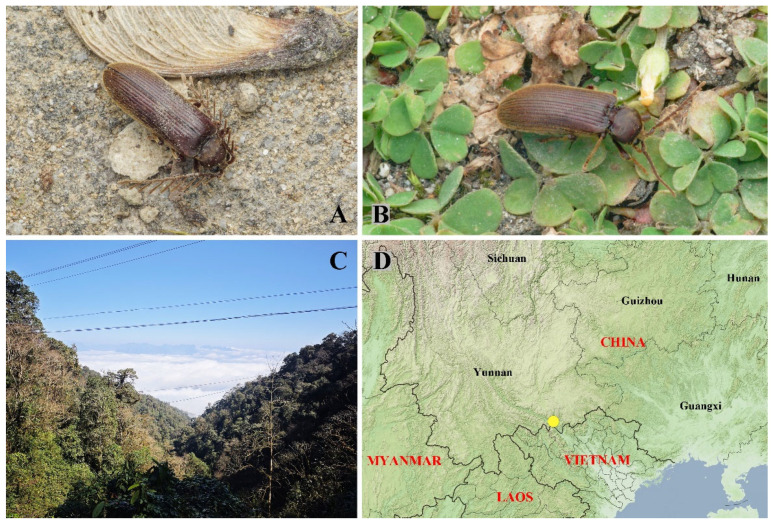
(**A**–**C**) Living individuals and habitat of *Cerophytum lii* **sp. nov.** from Mount Daweishan, Yunnan; (**A**) A living male attracted by light; (**B**) A living female attracted by light; (**C**) Environment of Mount Daweishan; (**D**) Distribution map of *Cerophytum* species in China (yellow dot indicates Mount Daweishan, where *C. lii* sp. nov. and *Cerophytum* sp. were both discovered). (**A**–**C**) photographed by Yi-Teng Li.

Habitus photographs of *C. lii* sp. nov. (Figure 1) were taken using a Canon EOS RP + Mount Adapter EF-EOS R with a 100 mm F2.8 CA-Dreamer Macro 2 × lens (for Canon EF). Diagnostic characteristics were photographed with the same camera setup, using either a Laowa 25 mm F2.8 2.5–5 × Ultra Macro Lens (for Canon EF) (Figure 2) or a Mitutoyo M Plan Apo 10 × / 0.28 lens (Figure 3). Photographs of *Cerophytum* sp. were taken using a Canon D800 camera attached to a Canon MP-E 65 mm Lens (Figure 4A,B) or a microscope lens (Figure 4C–E). The distribution map (Figure 5D) was generated from QGIS 3.40.1-Bratislava. All figures were processed in Adobe Photoshop CC 2019.

### 2.4. Micro-CT Scanning and 3D Reconstructions

One male paratype specimen of *C. lii* sp. nov. (preserved in absolute ethanol) was selected for Micro-CT Scanning and 3D Reconstructions. The specimen was dehydrated through an ascending ethanol series (80%, 90%, 96%, 100%) [20]. To prevent deformation of internal structures during drying, it was then immersed in hexamethyldisilane (HDMS) for 12 h, followed by air-drying under ventilated conditions for 48 h to facilitate HDMS decomposition. The resting position of the specimen was scanned using a Scanco Medical μCT100 scanner (Scanco Medical Inc., Wangen-Brüttisellen, Switzerland) with the following settings: beam strength 45 kV 200 μA 9W, voxel size 3.3 μm, FOV: 10.137 mm, absorption contrast 360 steps, image 3072 × 3072, and 3384 obtained sections. Volume rendering and 3D reconstructions were conducted in Drishti 3.2 [21]. The .tiff stacks were imported into Drishti Import to produce a .pvl.nc file, which was then loaded into Drishti Paint. Segmentation of individual appendages was carried out after loading the .pvl.nc file in Drishti Paint, employing various tools. The exported files were imported into Drishti renderer for visualization purposes.

## 3. Results

### 3.1. Taxonomy

**Genus *Cerophytum* Latreille, 1806**

#### 3.1.1. *Cerophytum lii* Qiu & Ruan, sp. nov.

**Figure 6:**
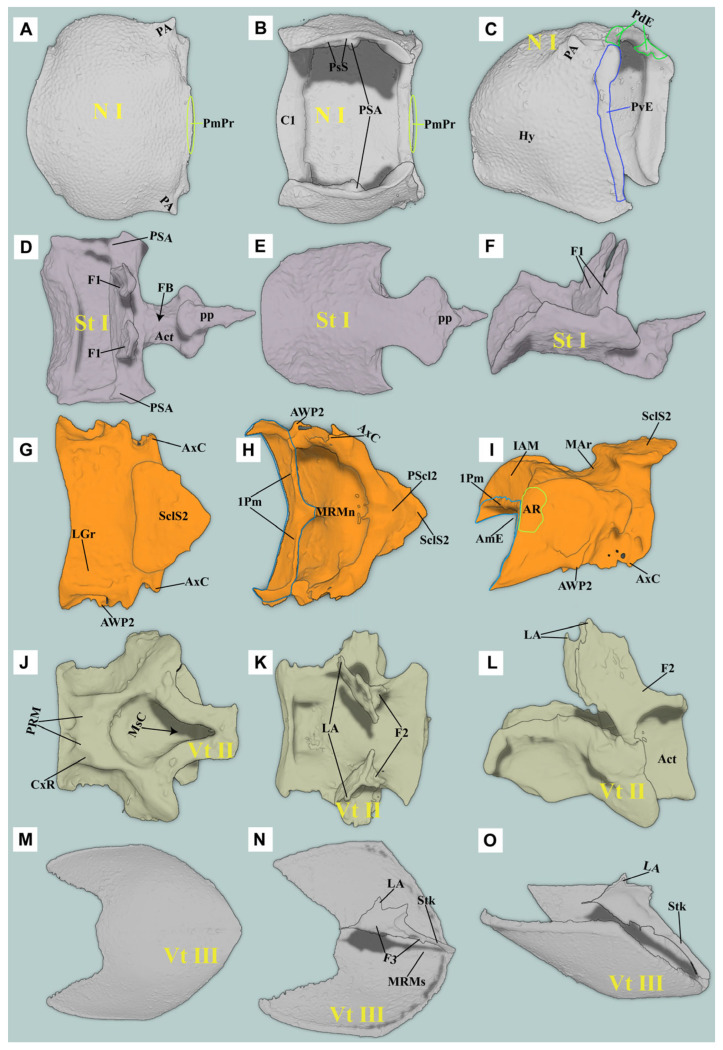
Three-dimensional reconstructions of the thoracic exoskeleton of *Cerophytum lii* **sp. nov.**: (**A**–**C**) Dorsal, ventral and lateral views of the pronotum; (**D**–**F**) Ventral, dorsal and lateral views of the prosternum; (**G**–**I**) Dorsal, ventral and lateral views of the mesonotum; (**J**–**L**) Ventral, dorsal and lateral views of the mesoventrite; (**M**–**O**) Ventral, dorsal and lateral views of the metaendosternite. For abbreviations see the “Materials and Methods” section.

**Figure 7:**
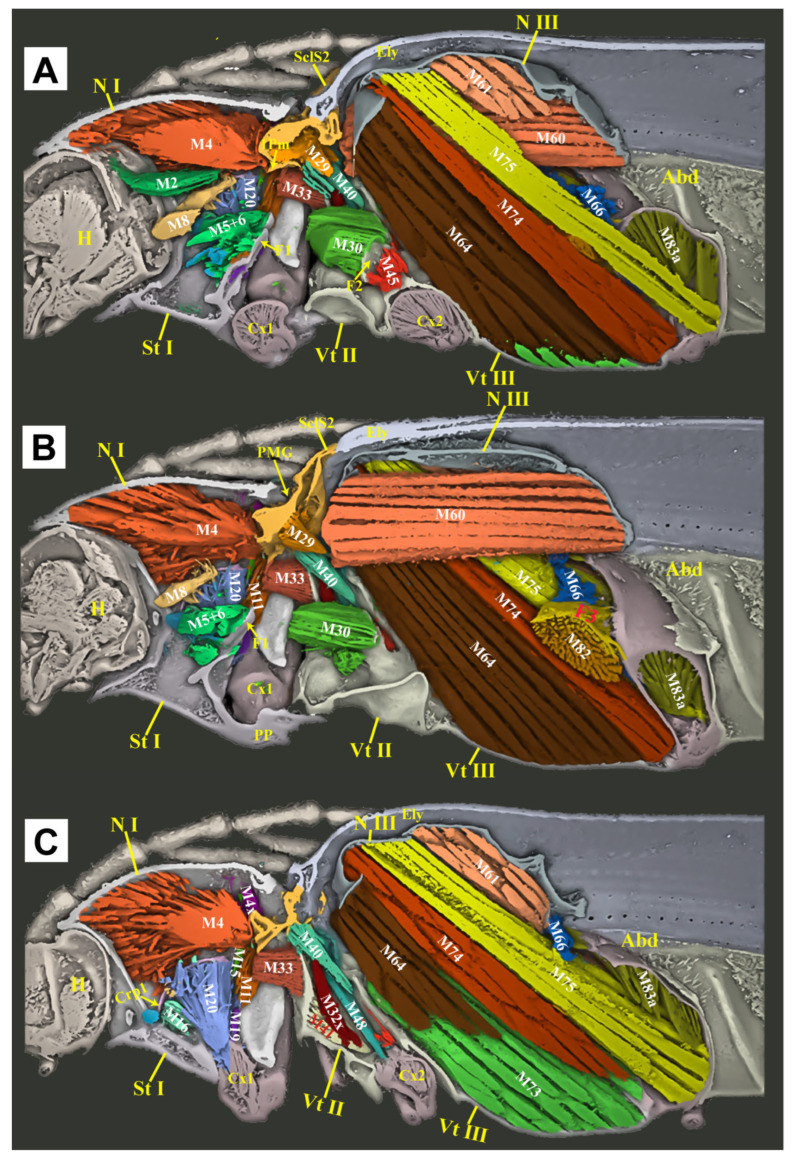
Three-dimensional reconstructions of the thoracic morphology of *Cerophytum lii* **sp. nov.**, lateral view. The model’s head is facing left; the exoskeleton is cut along the parasagittal and sagittal planes, to show the internal muscles: (**A**) Model is cut along the left 1/3 in the parasagittal plane; (**B**) Model is cut in the sagittal plane, muscles on the left 1/2 of the body are hidden; (**C**) Model is cut along the left 2/3 in the parasagittal plane, muscles on the left 2/3 of the body are hidden. For abbreviations see the “Materials and Methods” section.

**Figure 8:**
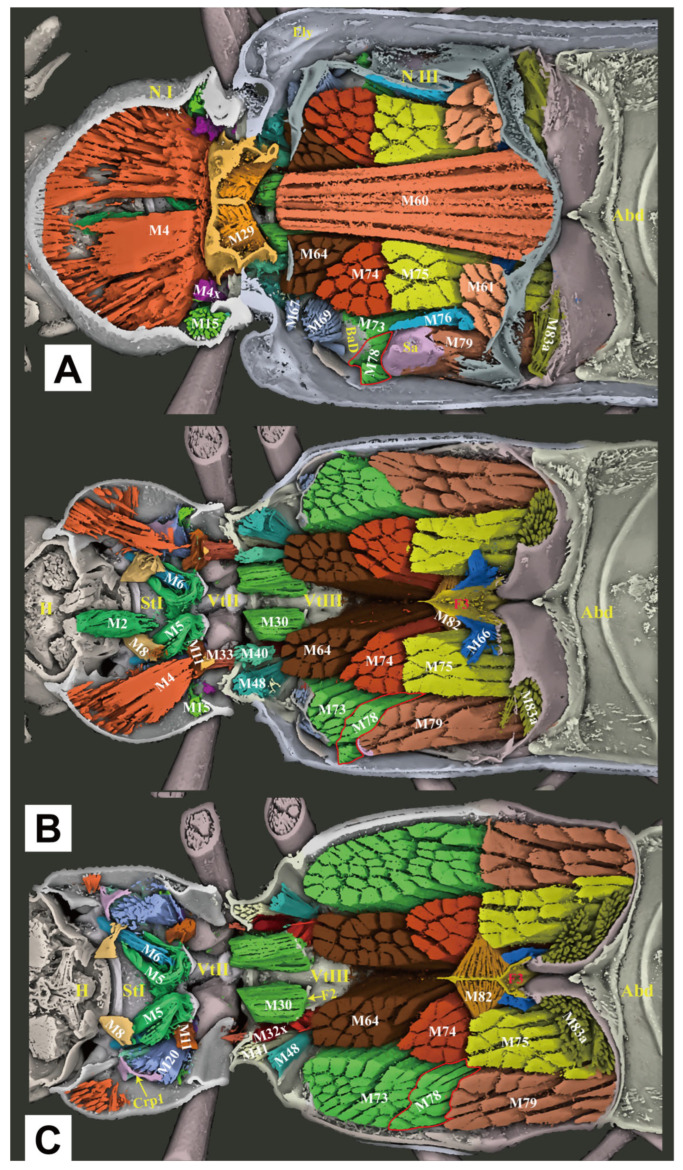
Three-dimensional reconstructions of the thoracic morphology of *Cerophytum lii* **sp. nov.**, dorsal view. The head of the model is facing left, and the model is cut in the frontal (coronal) plane at different layers to show internal musculature: (**A**) Model is cut along the dorsal 1/3 in the frontal (coronal) plane; (**B**) Model is cut in the frontal (coronal) plane, muscles on the dorsal 1/2 of the body are hidden; (**C**) Model is cut along the dorsal 2/3 in the frontal (coronal) plane, muscles on the dorsal 2/3 of the body are hidden. For abbreviations see the “Materials and Methods” section.

Figure 1, Figure 2, Figure 3, Figure 5, Figure 6, Figure 7 and Figure 8.

Zoobank: urn:lsid:zoobank.org:act:16C2EA0E-7D46-43B0-A4D7-0760DFA4A193

**Chinese common name.** 李氏树叩甲

**Type locality.** Mount Daweishan, Pingbian County, Honghe Prefecture, 2100 m, Yunnan Province, China.

**Type material.** Holotype: male (MYNU), Mount Daweishan [大围山], Pingbian County [屏边县], Honghe Prefecture [红河州], Yunnan Province, China, 2100 m, 23–24.I.2025, Yi-Teng Li [李奕腾] leg. Paratypes: 1 male (CYTL) and 1 female (MYNU), same data as holotype.

**Diagnosis.** Relatively large species in the genus with elongate body (body length 9.3–9.8 mm, body width 3.0–3.2 mm). Body uniformly reddish brown. Maxillary palpomere III 1.9 times longer than wide, with round apex. Antennae of male extending beyond middle of elytra, strongly pectinate from antennomere 3 to 10. Punctures on head and pronotum large, shallow, umbilicate, and dense; with intervals nearly absent. Scutellar shield triangular, wider than long. Elytra elongate, 2.6 times longer than wide, 0.8–0.9 times body length. Profemur with upper distal angle quadrate (Figure 2G, black arrow).

**Comparison.** This new species differs from all other known species of *Cerophytum* by its distinctly larger body (body length 9.3–9.8 mm, compared to 5.4–8.5 mm in other described species) and the quadrate upper distal angle of profemur (rounded in other species). The more slender elytra also distinguish the new species from all others (elytra 0.8–0.9 times the body length, compared to 0.6–0.7 times in other described species). Additionally, *C. lii* sp. nov. differs from *C. japonicum* and *C. convexicolle* by its strongly pectinate antennomere 3 (which is only triangularly serrate in the latter two species) [1,22,23].

**Description.** Male holotype (Figure 1A). Length 9.8 mm, width 3.0 mm, antennal length 7.5 mm, pronotum length × width = 2.0 × 2.5 mm, elytral length 7.8 mm. Body reddish brown, densely covered with fine yellow pubescence.

Eyes large, globose (Figure 2A). Interocular length 0.4 times head width. Area between antennal sockets strongly convex. Frontoclypeal region somewhat vertical, medially carinate. Mandibles simple, curved (Figure 2D). Maxillary palpomere III subtriangular, 1.9 times longer than wide, apex rounded (Figure 2J). Antennae reaching beyond middle of elytra, strongly pectinate from antennomere 3 to 10 (Figure 1A). Antennomere 1 robust, long, slightly curved; antennomere 2 conical, shortest; antennae 3 to 10 similar in length, gradually thinner. Pectinate portions from antennomere 3 to 10 progressively increase in length toward apex, each distinctly longer than its respective antennomere length. In antennomere 3, pectinate portion 1.6 times antennomere length; in antennomere 4, pectinate portion 1.8 times antennomere length; in antennomere 5–10, pectinate portion exceeds twice each antennomere length. Last antennomere longest, 6.7 times longer than wide, apex rounded (Figure 2H). Head with punctures dense, large, shallow, umbilicate; puncture intervals extremely narrow, sometimes almost absent. Small unpunctured area present at base of head medially.

Pronotum convex, wider than long, widest at distal half; 0.8 times as wide as elytra, 0.3 times elytral length. Hind angles of pronotum short, sharp, pointing laterally. Punctures large, shallow, umbilicate, intervals dense, almost connected; disc with denser punctures than other areas, posterior margin with three small unpunctured areas medially and laterally beside hind angles (Figure 2A).

Prosternum widest near middle. Prosternal sutures deeply grooved apically. Prosternal lobe with anterior margin convex (Figure 2C). Prosternal process in lateral view strongly developed at basal half, abruptly narrowed and curved dorsal at distal half, forming a prominent step. Apical half of prosternal process slender, with apex slightly curved dorsally, apex narrowly rounded (Figure 2F). Punctures on prosternal lobe large, dense, uneven-sized, intervals slightly wrinkled; prosternal punctures smaller toward prosternal process, medially with sparser punctures, intervals about one puncture diameter; punctures between procoxae much smaller than other parts, intervals about one puncture diameter medially, less than one laterally. Puncture size and density on hypomeron same as on middle part of pronotum. Mesoventrite with straight anterior margin in ventral view (Figure 2C). Metaventrite and abdomen with similar small, shallow punctures; punctures larger and denser at metaventrite base.

Profemur with longitudinal carina (Figure 2G, red arrow), upper distal angle quadrate (Figure 2G, black arrow). Tarsal claws serrate.

Scutellar shield triangular, densely punctate, 0.7 times longer than wide (Figure 2A).

Elytra elongate, 2.6 times longer than wide (Figure 1A). Sides subparallel, medially slightly narrowed, apices rounded. Humeri rounded. Striae formed by large oval punctures. Interstriae smooth, slightly wrinkled, with micropunctures. Subhumeral row of punctures between striae 8 and 9 absent. Hindwings fully developed (capable of flight).

Tergite VIII sub-oval, wider than long, apex narrowed (Figure 3A); tergite IX and X connate, semi-oval, apical portion asymmetrical (Figure 3C). Sternite IX elongate oval, 1.8 times longer than wide, apical portion setose, apex rounded (Figure 3B). Base of sternite IX and tergite IX fused.

Aedeagus (Figure 3D–K): median lobe about 4.3 times as long as wide, widest at base, apex narrowed, in lateral view, L-shaped (Figure 3J,K); phallobase as in Figure 3F–G, basal half widened, basal margin deeply concave; distal half of phallobase spoon-shaped, with base extremely narrowed and rod-like, apical portion enlarged, apex with deep incision; middle region of parameres with hook˗like structure, distal region membranous (Figure 3D,E,H,I).

Male paratype. Body length 9.3 mm; nearly identical to holotype male; the aforementioned small unpunctured areas on the posterior margin of pronotum are indistinct.

Female paratype (Figure 1B). Body length 9.5 mm; similar to males, but with shorter serrate antennae and slightly larger pronotum (Figure 2B). Antennae not reaching half of elytra (Figure 1B). Antennomere 3 elongate, 2.1 times longer than wide, antennomere 4–10 triangularly serrate, antennomere 11 elongate, 3.1 times longer than wide (Figure 2I). The aforementioned small unpunctured areas on the posterior margin of pronotum indistinct. Tergite VIII with apex rounded (Figure 3L); sternite VIII with apex rounded, spiculum ventrale 4.9 times longer than sternite VIII length (Figure 3M). Ovipositor short, coxite about 1/4 of total length, sides setose, styli cylindrical, attached almost apically (Figure 3N). Bursa copulatrix with pair of sclerotized structures, surface spinous (Figure 3N,O). Spermatheca present, curved and sclerotized (Figure 3P).

**Distribution.** China (South Yunnan) (Figure 5D).

**Ecology and bionomics.** This new species is phototactic. The specimens shown in Figure 5A,B were observed resting or crawling near a light source, to which they were attracted. Collections were made in January, a period when most beetles in China are relatively inactive.

**Etymology.** The species epithet honors the collector Yi-Teng Li, for his valuable support of this study.

**Remarks.** Costa et al. [1] noted that the upper distal angle of profemur is rounded in the genus *Cerophytum*; however, in this new species, it is somewhat quadrate. Nevertheless, all other characteristics of the new species align with the diagnostic characteristics of *Cerophytum* (e.g., frontoclypeal region with longitudinal carina, pronotum with hind angles produced laterally, row of punctures between striae 8 and 9 absent, profemur with longitudinal carina, etc.), firmly placing it within this genus.

#### 3.1.2. *Cerophytum* sp.

Figure 4

**Material examined.** 1 female (SZPT), Mount Daweishan [大围山], Pingbian County [屏边县], Honghe Prefecture [红河州], 2050 m, 29.V–3.VI.2021, Xin-Yuan Zhang & Hao Xu leg.

**Diagnosis.** Female (Figure 4A,B): body stout, length 10.4 mm, width 3.9 mm. Body dark brown, profemur dark brown, rest legs and antennae reddish brown. Antennae reaching middle of elytra, strongly serrate from antennomere 3 to 10 (Figure 4C). Punctures on pronotum large, shallow, umbilicate; distal half of pronotum with numerous punctures fused into grooves (Figure 4D). Pronotum wider than long, widest at distal half; 0.7 times as wide as elytra, 0.3 times elytral length. Elytra elongate, 2.6 times longer than wide, 0.8–0.9 times body length. Profemur with upper distal angle rounded (Figure 4E).

**Remarks.** This species is distinctly different from *C. lii* sp. nov. by the slightly larger body, different colorations and antennomere shapes, different proportions of pronotum and elytra, as well as the different shapes of profemur and punctures on pronotum. Due to only one female specimen being available, we recorded the species here for future studies.

#### 3.1.3. Key to Known Species of *Cerophytum* (Modified from Costa et al. [1])

1. 1. Antennomere 3 of male serrate; antennomere 11 of female broader, about 2.2 times as long as wide…………………………………………………………………………………………………………2

- Antennomere 3 of male pectinate; antennomere 11 of female slenderer, about 2.5–3.1 times as long as wide……………………………………………………………………….3

2. Antennomere 11 of male slenderer, about 4.3 times as long as wide (Japan and South Korea)……………………………………………………………………………*C. japonicum*

- Antennomere 11 of male broader, about 3.1 times as long as wide (western U.S.A.)…………………………………………………………………………*C. convexicolle*

3. Body larger (9.3–9.8 mm), elytra 0.8–0.9 times of body length; upper distal angle of profemur quadrate (China)…………………………………………………..*C. lii* sp. nov.

- Body smaller (5.4–8.5 mm), elytra 0.6–0.7 times of body length; upper distal angle of profemur rounded…………………………………………………………………..4

4. Antennomere 11 of male and female with well-marked preapical notch (Europe)…………………………………………………………………………….*C. elateroides*

- Antennomere 11 of male and female with preapical notch weak or absent (eastern U.S.A.)…………………………………………………………………………*C. pulsator*

### 3.2. Thoracic Morphology of Cerophytum lii *sp. nov.*

#### 3.2.1. Prothorax

**(1) Pronotum** (Figure 6A–C)

Pronotum (N I) small (compared to most species of clicking Elateridae), subquadrate in dorsal view; subtrapezoidal in lateral view. Lateral carina absent; hypomeron of pronotum well-developed. Anterior margin of the pronotum arcuate and slightly produced forward.

**Posteromedian part of the pronotum (PmPr):** almost absent. **Posterodorsal evagination (PdE):** weakly developed, with highly smooth ventral and posterior surfaces. **Posteroventral evagination (PvE):** well-developed, strongly sclerotized, internal surface highly smooth. **Posterodorsal groove (PGr):** almost absent or extremely weakly developed. **Posterior angle of the prothorax (PA):** extremely weakly developed, wedge-shaped, producing laterally.

**(2) Propleuron** (Figure 7C)

**Cryptopleuron (Crpl):** normally developed, disc-shaped with M16 and M20 attached.

**(3) Prosternum** (Figure 6D–F)

**Prosternal process (PP):** well-developed basally, weakly developed apically; well-sclerotized, wedge-shaped, acute apically; well extending posteriorly beyond procoxae. PP in lateral view: straight at base, ventral surface abruptly elevated at middle (forming a step), slightly curved apically. **The friction hold (FH):** absent. **Profurca (F1):** well-developed, strongly elongated and produced dorsally, fan-shaped, with M5, M6, M30, and M11 attached. Profurcal base (FB) well sclerotized. **Pronotosternal suture (PsS):** anterior part flexible; posterior part closely attached to the hypomeron by pronotosternal articulation (**PSA**). **Procoxa** (Figure 7: **Cx1**)**:** subglobose; coxal cavities open externally.

#### 3.2.2. Mesothorax

**(1) Mesonotum (N II)** (Figure 6G–I)

**Mesonotum (N II):** specialized, slightly saddle-shaped, moderately sclerotized. In lateral view, with middle part concave and arched ventrally (MAr), concavity depth reaching ~1/3 of vertical height of N II. **Anteromedian emargination of mesonotum (AmE):** weakly developed, V-shaped in dorsal view, making space for the insertion of M2. **Anterolateral region of mesonotum (AR):** extremely weakly developed, oblique and subhemispherical; surface highly smooth, dorsal margin conforming to posterodorsal evagination (PdE). **Prealar bridge of mesonotum (PaBr):** normally developed. **First phragma (1Pm):** weakly developed. **Median-arched area of the mesonotum (MAr):** weakly developed, forming a shallow fovea. **Mesoscutellum (Scl2):** normally developed; mesoscutellar shield (SclS2) raised above the surface of the mesonotum. **Yoke plate (YP):** weakly developed.

**(2) Mesopleuron**

Mesopleural wing process (PlWP2): weakly developed. Mesanepisternum (Es2) and Mesepimeron (Em2): well-developed.

**(3) Mesoventrite (Vt II)** (Figure 6J–L)

Mesoventrite deeply excavated ventrally; anterior margin straight. In lateral view, posterior part of the mesoventrite forms acetabula (Act) to accommodate mesocoxae. In dorsal view, anterior and lateral margins strongly raised. Anteroventral angle of mesoventral cavity (AVA) obtuse.

**Prosternal rest of the mesoventrite (PRM):** extremely weakly developed, only faintly traceable as an impression with smooth surface. **Mesoventral cavity (MsC):** weakly developed, forming a shallow oval cavity in ventral view, surface smooth and unsculptured.

#### 3.2.3. Metathorax

Its general form is not greatly different from *Campsosternus auratus* [13].

**(1) Metanotum (N III)**

Metanotum subtrapezoidal in dorsal view, 0.7 times longer than wide, surface smooth.

**(2) Metaventrite (Vt III)** (Figure 6M–O)

Metaventrite (Vt III) strongly sclerotized, quadrate in general shape. Metafurca (F3, also known as metaendosternite) well-developed, cruciform in structure.

#### 3.2.4. Thoracic Musculature

**(1) Prothoracic Musculature** (Figure 7 and Figure 8)

**M1** (Idlm2): *M. pronoti primus*. **O:** anteromedian part of the pronotum; **I:** dorsolateral part of the postoccipital ridge.

**M2** (Idlm1): *M*. *pronoti secundus*. **O:** median dorsal apex of the first phragma (1Pm) (uncertain, muscle ruptured in the scanned specimen); **I:** dorsolateral part of the postoccipital ridge.

**M4** (Idlm5): *M*. *pronoti quartus*. **O:** major area of the pronotum; **I:** anterolateral part of the first phragma (1Pm). M4 well-developed, but weaker than M60, M64, M74 and M75; significantly weaker than that of *Campsosternus auratus* [13].

**M4x** (Idlm5). **O:** posterior part of the pronotum (N I); **I:** prealar bridge of the mesonotum (PaBr).

**M5** (Ivlm3): *M*. *prosterni primus*. **O:** profurcal arm (F1); **I:** posterior tentorial arm of the head.

**M6** (Ivlm1): *M*. *prosterni secundus*. **O:** profurcal arm (F1); **I:** ventral part of the cervical membrane and the anterior cervical sclerite (Cv1).

**M7** (Idvm6): *M*. *dorsoventralis primus*. Absent or weakly developed (uncertain).

**M8** (Idvm8): *M*. *dorsoventralis secundus*. **O:** lateral part of the first phragma (1Pm) (uncertain, muscle ruptured in the scanned specimen); **I:** ventrolateral part of the postoccipital ridge.

**M10** (Idvm2, 3): *M*. *dorsoventralis quartus*. **O:** anterolateral part of prosternum (St I); **I:** dorsal part of the postoccipital ridge.

**M11** (Idvm10): *M*. *dorsoventralis quintus*. **O:** profurcal arm (F1); **I:** lateral part of the first phragma (1Pm).

**M15** (Idvm16, 17): *M*. *noto-coxalis*. **O:** posterolateral part of the pronotum (N I); **I:** process of the procoxa (Cx1).

**M16** (Ipcm4): *M*. *episterno-coxalis*. **O:** anterior part of the cryptopleuron (Crpl); **I:** process and rim of the procoxa (Cx1).

**M19** (Iscm2): *M*. *furca-coxalis*. **O:** profurcal arm (F1); **I:** process of the procoxa (Cx1).

**M20** (Ipcm8): *M*. *pleura-trochanteralis*. **O:** posterior part of the cryptopleuron (Crpl); **I:** trochanter of the proleg. Much stronger than that of *Campsosternus auratus* [13].

**(2) Mesothoracic Musculature** (Figure 7 and Figure 8)

**M28** (IIdlm1): *M*. *mesonoti primus*. **O:** posterior part of the first phragma (1Pm); **I:** dorsal part of the second phragma (2Pm).

**M29** (IIdlm2): *M*. *mesonoti secundus*. **O:** posterior part of the first phragma (1Pm); **I:** anterolateral part of the metathoracic prescutum (Prs3).

**M30** (Ivlm7): *M*. *mesosterni primus*. **O:** proforcal arm (F1); **I:** mesofurcal arm (F2).

**M32x** (IIdvm8?): *M*. *dorso-ventralis*. **O:** mesothoracic axillary sclerites (?); **I:** lateral inflected area of the mesoventrite (Vt II).

**M33** (IItpm2): *M*. *noto-pleuralis*. **O:** first phragma (1Pm); **I:** pleural arm of the mesopleuron (PlA).

**M36** (IItpm9): *M*. *pleura-alaris*. **O:** pleural arm of the mesopleuron (PlA); **I:** third axillary sclerite of the mesothorax (Ax3).

**M37** (IIspm2): *M*. *furca-pleuralis*. **O:** mesofurcal arm (F2); **I:** lower part of the pleural ridge of the mesopleuron (PlR).

**M40** (IIdvm4, 5): *M*. *noto-coxalis*. **O:** posterolateral part of the mesonotum (N II); **I:** posterior rim of the mesocoxa (Cx2).

**M41** (IIpcm4): *M*. *episterno-coxalis*. **O:** mesanepisternum (Es2); **I:** anterolateral rim of the mesocoxa (Cx2).

**M45** (IIscm4): *M*. *furca-coxalis lateralis*. **O:** mesofurcal arm (F2); **I:** posterolateral rim of the mesocoxa.

**M46** (IIscm2): *M. mesofurca-coxalis* posterior. Absent or weakly developed (uncertain).

**M48** (IIpcm6): *M*. *episterno-trochanteralis*. **O:** mesanepisternum (Es2); **I:** trochanteral tendon.

**(3) Metathoracic Musculature** (Figure 7 and Figure 8)

**M60** (IIIdlm1): *M*. *metanoti primus*. **O:** second phragma (2Pm) and the middle part of the metathoracic prescutum (Prs3); **I:** median lobe of the third phragma (3Pm-ML) and postnotum (Pn3). Much stronger than that of *Campsosternus auratus* (Ruan et al. 2022 [13]).

**M61** (IIIdlm2): *M*. *metanoti secundus*. **O:** middle part of the metascutum (Sct3); **I:** lateral process of the third phragma (3Pm-LP).

**M64** (IIIdvm1): *M*. *dorsoventralis primus*. **O:** median part and median ridge of the metaventrite; **I:** metathoracic prescutum (Prs3). Much stronger than that of *Campsosternus auratus* [13].

**M66** (IIIdvm8): *M*. *dorsoventralis tertius*. **O:** lateral arm of the metafurca (LA); **I:** lateral process of the third phragma (3Pm-LP).

**M67** (IIItpm2): *M*. *pleura-praealaris*. **O:** prealar sclerite (Pra); **I:** pleural ridge of the metapleuron (PlR).

**M69** (IIItpm3): *M*. *noto-basalaris*. **O:** lateral part of the metathoracic prescutum (Prs3); **I:** basalar disc (BaD).

**M71** (IIItpm7, 9): *M*. *pleura-alaris*. **O:** metanepisternum (Es3); **I:** a small sclerite in the membrane under the third axillary sclerite.

**M73** (IIIspm1): *M*. *sterno-basalaris*. **O:** lateral part of the metaventrite (Vt III); **I:** basilar disc (BaD).

**M74** (IIIdvm2): *M*. *noto-trochantinalis*. **O:** anterior part of the metascutum (Sct3); **I:** trochantinal disc. Much stronger than that of *Campsosternus auratus* (Ruan et al. 2022 [13]).

**M75** (IIIdvm4): *M*. *noto-coxalis anterior.* **O:** middle part of the metascutum (Sct3); **I:** inner surface of the metacoxa (Cx3). Much stronger than that of *Campsosternus auratus* [13].

**M76** (IIIdvm5): *M*. *noto-coxalis posterior*. **O:** lateral margin of the metascutum (Sct3); **I:** inner surface of the metacoxa (Cx3).

**M78** (IIIpcm3): *M*. *coxa-basalaris*. **O:** anterior margin of the metacoxa (Cx3); **I:** basilar disc (BaD).

**M79** (IIIdvm6): *M*. *coxa-subalaris*. **O:** inner surface of the metacoxa (Cx3); **I:** subalar disc (Sa).

**M81** (IIIscm1): *M*. *furca-coxalis anterior*. **O:** stalk of the metafurca (Stk); **I:** anteromesal rim of the metacoxa (Cx3).

**M82** (IIIscm4): *M*. *furca-coxalis lateralis*. **O:** ventral flange of the metafurca (VF); **I:** a process on the anterolateral rim of the metacoxa (Cx3).

**M83a** (IIIscm2): *M*. *metafurca-coxalis posterior*. **O:** dorsal surface of the lateral arm of the metafurca (LA); **I:** posterior rim of the coxa (Cx3).

**M83b** (IIIscm3): *M*. *metafurca-coxalis posterior*. **O:** ventral surface of the stalk of the metafurca (Stk); **I:** mesal part of the posterior rim of the metacoxa (Cx3).

**M85** (IIIscm6): *M*. *furca-trochanteralis*. **O:** lateral arm of the metafurca (LA); **I:** trochanteral tendon.

#### 3.2.5. Comparative Thoracic Morphology of *Cerophytum lii* sp. nov. and *Campsosternus auratus*

The thoracic exoskeletons of both species are largely homologous but differ in the degree of sclerotization and structural robustness. *C. lii* sp. nov., in comparison to *Campsosternus auratus*, exhibits a significantly reduced prothorax, a slenderer prosternal process (particularly in its distal half), weaker posterior pronotal evaginations (PdE and Pmpr), and a less sclerotized mesonotum with a shallower median arched area (MAr).

The thoracic muscle attachments are largely consistent between *C. lii* sp. nov. and *Campsosternus auratus*, differing primarily in minor structural modifications. Specifically, in *C. lii* sp. nov. (compared to *Campsosternus auratus*), muscles M1 and M7 are significantly more gracile and less developed, while M4 exhibits reduced strength relative to M60, M64, M74, and M75—a notable contrast to *Campsosternus auratus*, where M4 is the most robust thoracic muscle and substantially stronger than those same comparator muscles.

Other minor morphological differences include the following characteristics: in *C. lii* sp. nov., muscles M10 and M20 are stronger, while M28 is significantly weaker and M30 is relatively well-developed. Muscles M45 and M46 are greatly reduced, appearing filiform and nearly absent. Additionally, M67 and M71 are more poorly developed—subconical and diminutive—whereas M76 is robust and exhibits a compressed cuboid shape. In contrast, M78 is weaker and closely associated with M73, rendering the two difficult to distinguish. All remaining thoracic muscles are congruent with those of *Campsosternus auratus*.

## 4. Discussion

### 4.1. Taxonomy and Bionomics

Cerophytidae is rare in collections, with Asian species being even scarcer than Neotropical ones [1]. To date, only one species, *Cerophytum japonicum*, has been recorded from Japan [22,23], with its range later extended to South Korea [9]. A tentative record of *Cerophytum* aff. *japonicum* has also been reported from the Russian Far East (Primorsky Krai) [10]. However, despite China’s vast territory, no specimens of Cerophytidae have been recorded in this country until this work. In this study, we present the first record of Cerophytidae from China, with two species of the genus *Cerophytum* discovered at the same locality (both collected at around 2050–2100 m on Mount Daweishan). Interestingly, although these two species are found almost in the same region, they seem to have completely different periods of activity. The new species, *C. lii* sp. nov., occurs “off-season” in January, while another undescribed species appears during the typical insect activity period (May–June). Both species were collected using one of the standard methods for Cerophytidae, light trapping, as is commonly used for this family [1,14]. It is worth noting that Mount Daweishan in Yunnan is one of China’s most renowned insect survey sites, where entomologists have conducted extensive specimen collections and discovered many interesting new species. However, Cerophytidae seems to have received relatively little attention, as no specimens of this family had been recorded in this region prior to this study. This may suggest that while Cerophytidae can be collected using conventional methods, such as light trapping, their activity period might be short, or they could be less attracted to light, leading to low capture rates. Regardless, the discovery of two *Cerophytum* species at the same location but in different seasons indirectly suggests that the diversity of this family may be greatly underestimated and that more interesting new species may be discovered in the future.

### 4.2. Functional Morphology

Although the clicking movement of *C. lii* sp. nov. has not been experimentally studied, its morphology suggests functional clicking ability, evidenced by well-developed M2 and M4 muscles (the primary clicking actuators) and a specialized saddle-shaped mesonotum with a pronounced median-arched area (MAr). We propose that this mesonotal structure functions analogously to that in *Campsosternus auratus*, facilitating elastic energy storage and release during clicking.

Although *C. lii* sp. nov. possesses all essential clicking-related muscles and sclerites, these structures are markedly less developed compared to *Campsosternus auratus*. Specifically, *C. lii* sp. nov. exhibits: (1) a significantly smaller prothorax, (2) a weakened prosternal process (PP) and mesonotum, (3) a reduced M4 muscle, (4) less pronounced posterodorsal pronotal evaginations (PdE and PmPr), and (5) an obsolete prosternal rest of the mesoventrite (PRM). Notably, the friction hold (FH), a critical structure for effective clicking in most Elateridae, is absent in *C. lii* sp. nov. Collectively, these morphological reductions suggest that *C. lii* sp. nov. likely produces less explosive clicks than *Campsosternus auratus*. Another notable morphological feature is the reduced posterior angles (PAs) of the pronotum in *C. lii* sp. nov. Since these structures are not functionally involved in the clicking mechanism, their reduction appears unrelated to the species’ less explosive clicking capability.

*C. lii* sp. nov. exhibits distinct metathoracic muscle development patterns compared to *Campsosternus auratus*. In *C. lii* sp. nov., muscles M60, M64, M74, and M75 are exceptionally well-developed, while M4 is comparatively weaker than these muscles. This contrasts sharply with *Campsosternus auratus*, where M4 is the dominant thoracic muscle, significantly more robust than M60, M64, M74, and M75. The hypertrophy of M60 and M64 in *C. lii* sp. nov. suggests enhanced flight capability relative to *Campsosternus auratus*.

The well-developed M74 and M75 muscles (walking muscles sensu Larsén [15]) in *C. lii* sp. nov. indicate enhanced locomotor functionality compared to *Campsosternus auratus*. The longer tarsi and legs in *C. lii* sp. nov. also provide superior terrestrial running performance [24]. Collectively, these features suggest *C. lii* sp. nov. has evolved a combined flying and cursorial (fast-walking) adaptive strategy. In conclusion, while *C. lii* sp. nov. and *Campsosternus auratus* exhibit convergent evolution of clicking structures, *Campsosternus auratus* demonstrates superior jumping performance through three key adaptations: (1) an elongated pronotum providing increased spaces for the M4 muscle, (2) highly developed clicking muscles (particularly M4), and (3) optimized elastic energy storage mechanisms, including a saddle-shaped mesonotum (NII) and reinforced prosternal process (PP). Conversely, *C. lii* sp. nov. has evolved morphological specializations favoring rapid terrestrial locomotion and flight capacity, reflecting distinct ecological selective pressures.

## Data Availability

The original contributions presented in this study are included in the article.
